# Development of Fortified Breads Enriched with Plant-Based Bioactive Peptides Derived from the Chia (*Salvia hispanica* L.) Expeller

**DOI:** 10.3390/foods12183382

**Published:** 2023-09-09

**Authors:** Brenda Ozón, Juliana Cotabarren, Florencia R. Geier, M. Paula Kise, Javier García-Pardo, Mónica G. Parisi, W. David Obregón

**Affiliations:** 1 Departamento de Ciencias Biológicas, Centro de Investigación de Proteínas Vegetales (CIProVe), Facultad de Ciencias Exactas, Universidad Nacional de La Plata, 47 y 115 s/N, La Plata B1900, Buenos Aires, Argentina; brendaozon@gmail.com (B.O.); cotabarren.juliana@biol.unlp.edu.ar (J.C.); florgei.94.fg@gmail.com (F.R.G.); davidobregon@biol.unlp.edu.ar (W.D.O.); 2 Departamento de Ciencias Básicas, Instituto de Ecología y Desarrollo Sustentable (INEDES, CONICET-UNLu), Universidad Nacional de Luján, Ruta 5 y Avenida Constitución, Luján B6700, Buenos Aires, Argentina; paulakise@yahoo.com.ar; 3 Institut de Biotecnologia i de Biomedicina (IBB) and Departament de Bioquímica i Biologia Molecular, Universitat Autònoma de Barcelona, Bellaterra, 08193 Barcelona, Spain

**Keywords:** antioxidant, bioactive peptides, bread, chia expeller, chia by-product, enzymatic hydrolysates, functional ingredient, plant-derived peptides, *Salvia hispanica* L.

## Abstract

By-products from the industrialization of oilseeds, particularly chia, can be sustainably used for the development of new functional products. In this work, wheat breads supplemented with up to 10 mg of chia expeller hydrolysate/g of flour were prepared, obtaining fortified breads with acceptability for consumption, according to a preliminary consumer research study based on an affective test employing a five-point hedonic scale of global acceptance. In this context, protein hydrolysates of the chia expeller were produced using Alcalase, reaching a degree of hydrolysis of 54.3 ± 1.6% with an antioxidant activity of 55.8 ± 0.4% after 6 h incubation at 25 °C in the presence of the enzyme. These peptides showed appropriate techno-functional properties and chemical compositions suitable for the further development of bakery products. Taken together, our approach and the development of a fortified bread with plant-based bioactive peptides provide a novel and eco-friendly alternative for the recovery of nutrients from agro-industrial waste. More importantly, these enriched breads could exert beneficial effects on human health by exploiting the antioxidant properties of functional peptides derived from the chia expeller.

## 1. Introduction

Wheat bread stands as a global staple food and is the main source of food and energy in most developing countries [1]. However, wheat-based diets are typically poor in essential amino acid content and in micro- and macronutrients; therefore, these essential amino acids and micro- and macronutrients must be incorporated through other dietary sources. Moreover, with the constant increase in the human population, food production must increase substantially to meet future demands. In this context, the incorporation of plant-based functional ingredients in wheat bread formulations has been shown to be an ideal approach to improve its organoleptic and nutritional properties [2,3,4,5].

Chia (*Salvia hispanica* L.) is a traditional crop native to Central and Southern Mexico and Guatemala, and nowadays, it is a cultivated plant across the landscapes of South and Central America [6,7]. Notably, the oil extracted from chia seeds has emerged as an outstanding reservoir of α-linolenic acid and phytosterols, surpassing many other vegetable oils in their concentrations. Of remarkable significance is the abundant presence of α-linolenic acid, constituting the predominant component of the fatty acid composition within chia seeds (approximately 60% of the total) compared with the lower proportion of linoleic acid (only about 10%) [6]. Moreover, chia seeds can be considered as a “functional superfood” because they contain a high concentration of protein (15–26%) of a good nutritional quality and digestibility and a good balance of essential amino acids that are higher than most grains, especially due to the high content of cysteine and methionine amino acids [8,9,10]. Another important characteristic of chia seeds is the absence of gluten, which makes it more desirable to coeliac patients. It also helps to increase the satiety index and prevent cardiovascular diseases, inflammatory and nervous system disorders, and diabetes, along with contributing to human nutrition [11].

A possible approach to increase the amino acid content and the nutritional quality of wheat bread is to incorporate food processing by-products [12,13]. It is worth mentioning that between a third and a quarter of food is lost or wasted annually along the food chain. This is also the case in the industrialization of chia seeds, in which the production of cold-pressed chia oil results in large amounts of protein-rich by-products. Among them, the chia expeller can be processed to yield functionally derived peptides that can be effectively used for the development of high value-added food products [13,14]. Typically, these bioactive peptides are inactive when bound inside the parent protein, and they should be enzymatically activated to fully display its beneficial biological properties [14].

In this study, we attempted to develop a novel functional product from bread by incorporating bioactive peptides produced via the enzymatic hydrolysis of the chia expeller by-product. These peptides display antioxidant properties and are a high-nutritional-value component, improving the amino acid content in the final product. In this context, we also investigated whether the incorporation of chia expeller hydrolysates into the bread formulation may alter its functional and sensory properties. Taken together, our results demonstrate that the enrichment of bread fortified with these chia-derived peptides may be a useful strategy to improve the protein content of the bread product and simultaneously generate more economic profitability for the chia industry.

## 2. Materials and Methods

### 2.1. Materials and Reagents

Protease from *Bacillus licheniformis* (Alcalase), sodium chloride, tris (hydroxymethyl) aminomethane, sodium dodecyl sulphate (SDS), β-mercaptoethanol (βME), Coomassie Blue G-250, N,N,N′,N′-tetramethyl ethylene diamine (TEMED), 1,1-diphenyl-2-picrylhydrazyl (DPPH), 2,2′-Azino-bis(3-ethylbenzothiazoline-6-sulf,onic acid) diammonium salt (ABTS), o-phthaldialdehyde (OPA), and bovine serum albumin were purchased from Sigma-Aldrich (St Louis, MO, USA). Ingredients such as wheat flour, yeast, sugar, salt, milk, butter, and oil were purchased locally.

### 2.2. Obtention of the Chia Expeller Protein Hydrolysate

Chia expeller, provided by Greenborg S.R.L. (Lincoln, Buenos Aires, Argentina), was defatted and then grinded to produce some flour with a high protein content [4]. Chia expeller protein concentrate (PC) was obtained by mixing 40 g of defatted flour with 1000 mL of 0.01 M phosphate buffer, 0.1 M NaCl, pH 7.4, before the mixture was subjected to subsequent homogenization in a cool blender and, after 1 h at room temperature, stored at −20 °C until analysis. A fraction of the PC was subjected to heat treatment at 50 °C for 1 h (called “HT”) to evaluate the degree of hydrolysis (DH%) after Alcalase treatment.

The enzymatic hydrolysis of PC and HT (PCH and HTH) was performed with the food-grade microbial protease Alcalase (enzyme/substrate ratio of 0.3 U g^−1^). The reaction mixture was adjusted to pH 7.0 to achieve the optimal enzymatic activity and incubated at 25 °C for 2, 4, 6, 8, 10, and 24 h at 80 rpm in a shaker (PCH25 and HTH25 samples) or 50 °C for 1, 2, 3, 4, 5, 6, 7, and 8 h at 80 rpm in a shaker (PCH50 and HTH50 samples) (Figure S1). All samples were withdrawn in triplicate, and the hydrolysis was stopped at the selected intervals via heating at 85 °C for 15 min. The hydrolysates were then lyophilised and kept at 4 °C until use.

### 2.3. Characterization of Hydrolysates

#### 2.3.1. Determination of the Degree of Hydrolysis

The degree of hydrolysis (DH%) was estimated by using the o-phthalaldehyde (OPA) method [15]. The OPA reagent was prepared as described by Ozón et al. [16]. A total of 10 µL of the sample and 200 µL of the OPA reagent were mixed in a 96-well flat bottom plate. The assay was performed by triplicate, including a negative control using water instead of the sample, and the absorbance at 340 nm was recorded after 2 min of incubation. A Tecan Infinite M200 PRO spectrophotometer (Männedorf, Switzerland) was used for the determinations. The DH was calculated as follows:

DH% = h/htot × 100

where h is the number of hydrolysed bonds, and htot is the total number of peptide bonds per protein equivalent (which depends on the amino acid composition of the raw material). Complete hydrolysis of the sample (100%) was achieved by incubating the sample with 6 N HCl at 100 °C for 24 h.

#### 2.3.2. Evaluation of Antioxidant Activity

The antioxidant activity of the chia expeller hydrolysates was evaluated by using the ABTS+ method [17] with slight modifications. The ABTS•+ solution was prepared through reacting an aqueous ABTS solution (5 mL) and potassium persulfate (K_2_S_2_O_8_) solution in a 2:1 ratio. After storage in the dark for 16–17 h, the blue–green radical cation solution was further diluted in phosphate buffer (pH 7.5) until its absorbance reached 1 ± 0.01 AU at 734 nm. The antioxidant compound content in PC, HT, and the hydrolysates were analysed by adapting the method to a 96-well flat bottom plate in a Tecan Infinite M200 PRO spectrophotometer (Tecan Group Ltd., Männedorf, Switzerland). A total of 190 μL of diluted ABTS•+ solution was added to a 10 μL sample or Trolox standard according to Ozón et al. [6]. The mixture was incubated for 10 min at room temperature in dark conditions, and after that, the absorbance was measured at 734 nm. ABTS+ radical scavenging activity was estimated as follows:

$$ABTS\;radical\;scavenging\;activity\;\left(\%\right)=100-100\times \frac{A1-A2}{A0}$$

The absorbance of the control without sample was expressed as A0; the absorbance in the presence of the sample and ABTS•+ and the absorbance of the sample blank without ABTS•+ was expressed as A1 and A2, respectively.

To evaluate the antioxidant capacity of the breads, 1 g of ground bread crumbs were homogenised in 5 mL of a phosphate buffer (0.1 M, pH 7.0). The suspensions were homogenised in a vortex mixer for 2 min and centrifuged at 7000× *g* for 10 min, and the supernatant from each sample was evaluated via the ABTS method, as previously described [17].

#### 2.3.3. Chemical and Techno-Functional Properties of the Chia Expeller Hydrolysates

PC hydrolysates produced by Alcalase hydrolysis at 25 °C for 6 h (PCH25,6) were selected for subsequent analysis. The moisture (AOAC 934.01), ashes (AOAC 942.05), lipids (AOAC 920.39), dietary fibre (AOAC 985.29), and protein (micro-Kjeldahl method, AOAC 984.13) contents of the raw materials were determined according to Association of Official Analytical Chemists (2000) protocols [18], and the carbohydrate content was obtained by subtracting 100 from the sum of other components.

Water retention capacity (WRC) and oil retention capacity (ORC) were determined according to Chau et al. [19] with modifications. Thus, 0.5 g of PCH25,6 were homogenised with 5 mL of water/oil via vortex for 2 min and centrifuged at 2200× *g* for 30 min. Water/oil on the supernatant was removed, and the remaining fibrous suspension was weighed to determine the water/oil weight gain. The WRC/ORC was expressed as grams of water/oil retained per gram of sample, considering oil density as 0.92 g/mL.

$$WRC\;or\;ORC=\frac{final\;weight-initial\;weight}{initial\;weight}$$

Water adsorption capacity (WAC) was determined according to Chen et al. [20]. A total of 1 g of PCH25,6 was placed in a microenvironment of 98% relative humidity in equilibrium, generated by placing 20 mL of saturated potassium sulphate saline solution in a hermetically sealed glass container at 25 °C. The sample was incubated until it achieved a constant weight, reporting this capacity as the weight gain expressed in grams of water per gram of dry sample.

$$WAC=\frac{g\;of\;adsorbed\;water}{g\;of\;CEH}$$

Emulsifying activity (EA) and emulsion stability (ES) were determined according to Chau et al. [19]. To evaluate EA, 100 mL of distilled water were added to 2 g of hydrolysates, and the mixture was homogenised at 11,000 rpm. Then, 100 mL of refined corn oil (density = 0.92 g/mL) were added and homogenised again at 11,000 rpm for 3 min in a disperser (Ultra-Turrax^®^, T25 Basic, IKA^®^, Werke, The Netherlands). The samples were placed in graduated centrifuge tubes (50 mL) and centrifuged at 170× *g* for 10 min, and the volume of the remaining emulsion was measured to calculate EA as follows:

$$EA=\frac{volume\;of\;remanent\;emulsion\;(mL)\;\times \;100}{initial\;volume\;of\;emulsion\;(mL)\;}$$

ES was determined by heating the obtained emulsions at 80 °C for 30 min. The samples were cooled to room temperature and centrifuged at 170× *g* for 10 min. ES was expressed as the ratio of the remaining emulsion volume (mL) with respect to the original volume of the emulsion.

$$ES=\frac{volume\;of\;remanent\;emulsion\;(mL)\;\times \;100}{initial\;volume\;of\;emulsion\;(mL)}$$

### 2.4. Ingredients and Breadmaking Procedure

Wheat breads were prepared with the addition of different PCH:flour ratios (i.e., 1, 3, 5, and 10 mg of PCH25,6/g flour (Table S1) using the N° 11 program of a commercial bread oven (ATMA Easy Cook, HP4031E). Wet ingredients were first added to the recipient, and then dry ingredients were placed on the top. The mixture was homogenised for 15 min at medium speed, fermented at 30 °C for 110 min with 80% relative humidity, and cooked for 50 min at 220 °C. After 12 h, the breads were sliced for further analysis.

### 2.5. Technological and Physicochemical Parameters

#### 2.5.1. Specific Volume Determination

Specific volume (SV) was determined according to the millet seed displacement method [21]. Millet seeds were poured into a container of known volume until the bottom was covered. The test bread was then placed inside the container, followed by more millet seeds, which were levelled across the top with a spatula. The displacement of the millet seeds that were not required to fill the container were measured in a graduated cylinder and used to express the specific volume.

#### 2.5.2. Colour Analysis

The breads were cut into 2 cm wide slices, and bread colour analysis was performed using a colour spectrophotometer (HunterLab MiniScan EZ, Reston, VA, USA) according to the colour system in the L*, a*, and b* space defined by CIE-L*a*b* [22]. Calibration with black and white tiles was performed before colour measurement.

The colour appearance of bread slices was quantified using three-dimensional colour spaces: lightness, colour, and hue. Lightness is described as the brightness of a surface, and colour indicates the intensity of the colour content (light green, dark green, etc.). Hue is the visual perception according to which an area appears to be similar to one of the colours: red, yellow, green, or blue. To calculate the hue, the CIE L*a*b* (CIELAB) colour space is taken into account, where the hue angle is calculated as h = arctan (b/a), between 0 and 360 degrees, corresponding to the hue circle, where (a) is the red–green component and (b) is the yellow-bluish component. In this way, h = 0 approximates the appearance of red object surfaces; h = 90 to the yellow ones, h = 180 to the green ones, and h = 270 to the blue ones. Colour difference values (ΔE), which measure the changes in colour of the total colour differences, were calculated as previously described [23] by using the following equation:

$$\sqrt{{∆L2+∆a}^{2}+{∆b}^{2}}$$

#### 2.5.3. Texture Profile Analysis

The textural properties of the fortified breads were determined on three central slices (2 cm thickness) using a TA-XTplus Texture Analyser (Stable Micro Systems, Surrey, UK) with a 36 mm cylindrical probe (p/36 R) according to the 74-09 methodology proposed by the Approved Methods of the American Association of Cereal Chemists (AACC) [21]. The pre-test speed, test speed, and post-test speed values were 1, 5, and 5 mm/s, respectively, with a trigger force of 5 g. The data were measured as force (N) versus time (s) curves. The hardness (g), adhesiveness (g.s), resilience (%), cohesiveness, and springiness (%) were recorded as well. Gumminess (G) was calculated as G = hardness × cohesiveness, and chewiness (Ch) was calculated as Ch = gumminess × springiness.

#### 2.5.4. Sensory Evaluation

The sensory analysis of freshly baked bread samples (CB and B10) involved 37 untrained panellists aged between 20 and 60 years who were regular consumers of wheat bread. This panel consisted of 18 women and 17 men, including undergraduate and graduate students, as well as faculty members from the Faculty of Exact Sciences at the National University of La Plata in La Plata, Argentina.

To assess the organoleptic characteristics of the breads, the panellists participated in a descriptive sensory analysis panel. The breads were baked the day before the evaluation. On the day of the evaluation, the untrained panellists were randomly divided into groups of nine people. These groups were accommodated in a room that was equipped with individual cabinets and specifically arranged for the sensory test. The panellists received basic instructions regarding the evaluation procedure, which was conducted in a single day. During the sensory evaluation, the bread samples (CB and B10) were presented as 2 cm cubes that were placed on different dishes, each labelled with random three-digit numbers. Water was provided for rinsing between tasting each sample.

Following the instructions, training, and discussions related to product-oriented affective testing, each panellist received a rating form score sheet. This score sheet was based on a five-point hedonic scale for assessing the overall acceptance of the bread. The scale ranged from 5, indicating “I liked it very much”, to 1, indicating “I disliked it very much”. Additionally, panellists performed a preference test where they selected their preferred bread out of all of the options.

The untrained panellists also performed a triangular test. This test is useful for evaluating overall bread differences between control samples and upon supplementation with the chia expeller-derived peptides. To perform the evaluation, three cubes were presented in random order: (i) CB-CB-B10, (ii) CB-B10-CB, (iii) B10-CB-CB. After sensory evaluation, the panellists opined on the different tastes of the bread cubes, and their answers were interpreted as “right”, “wrong”, or “do not know”.

The results were interpreted according to the relationship between the number of positive judgments and the total number of judgments. A significant difference between the samples at the corresponding level of probability was considered when the number of positive judgments was greater than or equal to the value of the two-sided table [24].

### 2.6. Statistical Analysis

Statistical analyses (ANOVA) were performed using GraphPad Prism (v9.4.1, GraphPad Software Inc., San Diego, CA, USA, 2012). All experiments were conducted in triplicate of two independent experiments. Data are expressed as the mean ± standard deviation, and significant differences were determined by using Tukey’s post hoc test (*p* < 0.05).

## 3. Results and Discussion

### 3.1. Enzymatic Hydrolysis of Chia Expeller Using Alcalase

**Figure 1:**
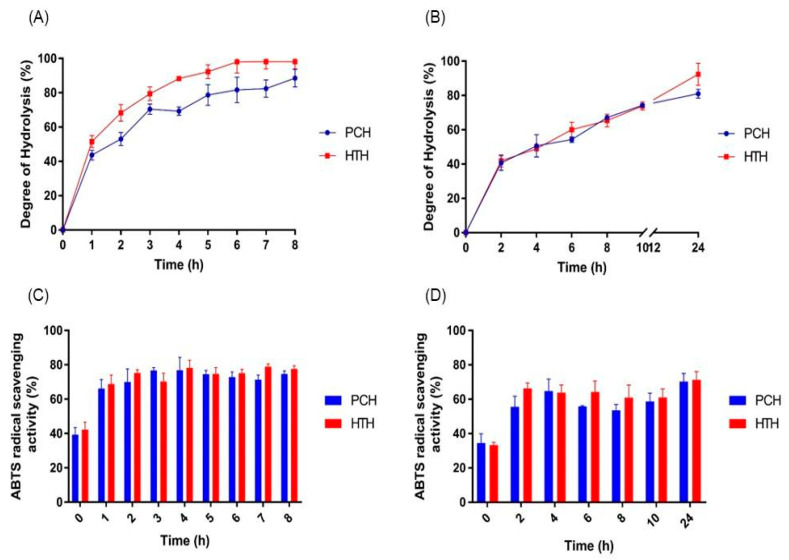
Characterization of chia expeller hydrolysates. Degree of hydrolysis (DH%) for enzymatic hydrolysis with Alcalase at 50 °C (**A**) and 25 °C (**B**) carried out using the OPA method. (**C**,**D**) ABTS radical cation decolorization assay to improve antioxidant activity for Alcalase hydrolysis at 50 °C (**C**) and 25 °C (**D**). In (**A**–**D**), the abbreviations are as follows: PCH, chia expeller protein concentrate hydrolysate; HTH, Heat-treated chia expeller protein concentrate hydrolysate.

Defatted chia expeller is a by-product with a high protein content of about 37% [14]. The optimization of the enzymatic hydrolysis of the chia expeller protein concentrate (PC) and the heat-treated sample (HT) was carried out to find the optimal digestion conditions. As observed in Figure 1, when the enzymatic hydrolysis was carried out at 50 °C, a gradual increase in the degree of hydrolysis (DH) was observed as the incubation time increased in both samples (PCH and HTH), reaching a DH of 70% after 3 h of incubation (Figure 1A). No significant increases in DH were observed at longer times, reaching 90% after 8 h, indicating a certain degree of enzyme inactivation when the samples were incubated at this temperature. However, when the enzymatic hydrolysis was carried out at 25 °C, the incubation time required to reach the same DH as in the test at 50 °C was longer, achieving 70% DH after 10 h of incubation for both samples (Figure 1B). Furthermore, by maintaining the process for 24 h, DH only increased up to 80%. Under our experimental conditions, the faster Alcalase hydrolysis of chia proteins was achieved at 50 °C incubation [16,25]. This observation aligns with the optimal temperature range for Alcalase activity, which typically falls between 50 and 65 °C [26].

Additionally, we also evaluated the effect that thermal pre-treatment had on the samples. Interestingly, thermally treating the samples at 50 °C for 1 h before the hydrolysis process (HTH) increased the DH by 10% in the first 4 h when the hydrolysis was carried out at 50 °C, suggesting that thermal treatment can also change substrate accessibility for hydrolysis due to substrate unfolding. No significant differences were observed between the DH obtained at higher hydrolysis times. On the other hand, when the hydrolysis was carried out at 25 °C, no important differences were observed in the resulting DH for both the thermally treated samples and the PC.

Next, we evaluated the antioxidant activity of the chia expeller protein concentrate hydrolysates (PCHs) and the corresponding heat-treated Alcalase hydrolysates (HTHs) produced at 50 °C, which was found to be the faster digestion condition. As shown in Figure 1C, the antioxidant capacity was similar for both sample groups (PCHs and HTHs) and was obtained after 2 to 8 h of hydrolysis, with values in the range 65–80%. Furthermore, the antioxidant activities produced by the chia expeller samples digested at 25 °C were also comparable (Figure 1D). When we compared the antioxidant activity of the hydrolysates obtained at 25 °C to those obtained at 50 °C, the values obtained after 2 h of hydrolysis at 50 °C were equivalent, which is in agreement with the DH values obtained. These results demonstrated the possibility of producing hydrolysates with Alcalase at room temperature, since they only require a longer hydrolysis time to obtain the same antioxidant effect as at 50 °C. Furthermore, no significant differences were observed in DH and the antioxidant activity when using PC and HT as substrates. Thus, we selected the PCH sample produced after 6 h at 25 °C (PCH25,6) for further characterization since this treatment showed a DH of 54.3 ± 1.6% and a high antioxidant activity (55.8 ± 0.4%).

### 3.2. Physicochemical and Techno-Functional Properties of the Chia Expeller Hydrolysates

The techno-functional properties of hydrolysates are important factors that need to be considered when developing novel ingredients as food additives [27]. For this reason, the proximal composition and the techno-functional properties of the hydrolysates (PCH25,6) produced in this study were characterised (Tables S2 and S3). As shown in Table S2, a protein content of 19.5% was determined for the PCH25,6 sample. This is slightly higher than the protein content reported in the literature for the chia seeds (16.5%, according to the USDA National Nutrient Database for Standard Reference) [28].

As expected, the total lipid content found in the PCH25,6 sample derived from the defatted expeller was dramatically reduced to 1.6%, a value that is substantially lower compared to the reference values for the chia seeds (30.7% of lipid content). Moreover, the ash content was higher compared to the values found in chia seeds (4.8% compared to 33%, respectively). The amount of assimilable carbohydrates were found to be 5.9%, and the soluble fibre content stayed in the range of 30–35%, with both values being comparable to those commonly found in the chia seeds (7.7% and 34.4%, respectively). Importantly, the protein content observed for PCH25,6 suggests that the chia expeller hydrolysate maintains protein and soluble fibre contents comparable to those found in the chia seeds, which suggest that chia expeller hydrolysates are excellent candidates for use as dietary supplements.

Extensive hydrolysates with a DH higher than 10% are commonly used for the generation of functional foods, including hypoallergenic and bioactive foods. In this study, we obtained PCH25,6 derived from the chia expeller, which has the particularity of containing a high protein and fibre content. In agreement with this, the Water Retention Capacity (WRC) of this was found to be 1.92 g H_2_O/g sample (Table S3). This value is in the same range to those observed in previous studies for chia seed protein isolates obtained via three distinct drying methods [29] and from flour obtained via solvent extraction or by pressing seeds [30]. In contrast, the oil retention capacity (ORC) of PCH25,6 was determined to be 2.36 g oil/g hydrolysate. This value is slightly increased compared to that reported by Capitani et al. [30], presumably due to the balance between the release of hydrophobic groups and the decrease in the molecular mass and release of ionizable groups, which probably have a negative effect on the ORC. Finally, the emulsifying activity of PCH25,6 was also determined. As shown in Table S3, we found values reported by Villanueva-Lazo et al. [31] for PCH25,6 that were slightly lower for the Alcalase hydrolysates derived from chia seeds (DH: 36.2%) than that reported for a defatted meal [30]. It is widely accepted that protein hydrolysis increases the emulsifying activity of food samples, as this has been previously reported for samples of corn, lupine, soybean, rice bran, pumpkin, oats, sesame, chickpea, peanut, rapeseed, and potato, among others [32].

### 3.3. Technological Parameters of the Fortified Breads Enriched with Functional Peptides Derived from the Chia Expeller

Plant-based proteins represent a good alternative to animal proteins in the production of functional peptides due to their low cost and ease of large-scale production. As described above, we have obtained chia hydrolysates produced via the enzymatic digestion of the chia expeller with Alcalase (i.e., PCH25,6). The resultant chia expeller hydrolysates displayed excellent values of emulsifying activity and water and oil retention capacities and an adequate proximal composition. Based on these results, breads enriched with different amounts of PCH25,6 (i.e., 1, 3, 5, and 10 mg of chia expeller hydrolysate/g of wheat flour) were formulated and analysed.

#### 3.3.1. External Analysis: Crust Colour

**Figure 2:**
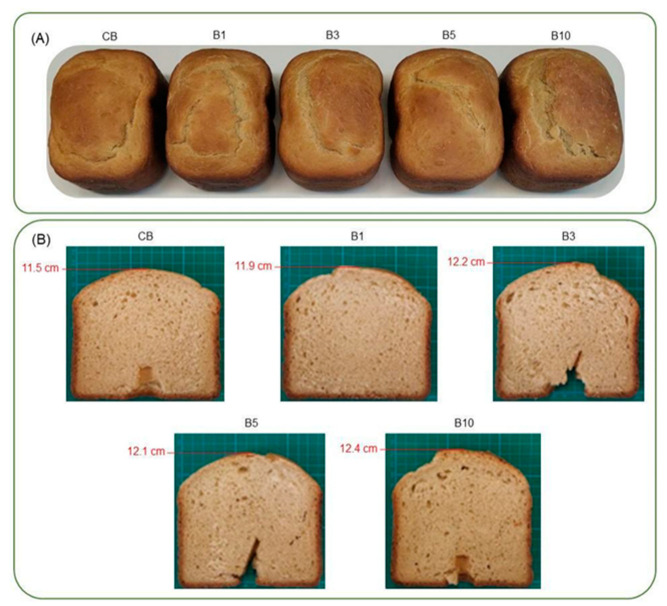
Photographs of wheat bread supplemented with chia expeller functional peptides. (**A**) Photographs of whole bread loaves. (**B**) Photographs of central transversal slices of each loaf positioned on a graduated cutting board. Bread slices produced with wheat flour (CB) or different blends of wheat flour via chia hydrolysates (B1, B3, B5 and B10).

The surface browning of bread is an indicator of bread quality. As shown in Figure 2A, the crust colour of breads containing increased concentrations of the chia expeller hydrolysates did not show significant differences compared to the bread made with wheat flour (control). In a previous study, Xing et al. [33] attributed a change in crust colour to a higher content of amino acids (specially lysine), which react with carbonyl compounds (reducing sugar in the Maillard reaction) during baking. We believe that the lack of darkening of the crust colour in our study is in agreement with the overall low lysine content (0.97 g/100 g) found in chia proteins [28].

#### 3.3.2. Alveolate and Specific Volume Analysis

The specific volume and the alveolate of bread crumbs are common indices of quality as they reflect the ability of the dough to occlude air. Regarding the alveolate, bread slices were scanned, and a qualitative analysis of images was performed (see Figure 2B). The addition of increasing amounts of hydrolysates revealed a positive effect on the formation of alveoli, with larger alveoli being observed in the breads supplemented with 3, 5, and 10 mg of hydrolysate per gram of flour than those observed for the control bread (CB). In addition, a considerable increase in height was also observed in the supplemented samples, showing an increase of 1 cm compared to the CB.

Interestingly, the specific volume was not significantly affected by the level of wheat flour substitution (Table 1). Only slightly higher values were recorded for the breads fortified with the chia hydrolysates (B10, B5, and B3, respectively), with mean values of 2.27 ± 0.05, 2.29 ± 0.08 and 2.25 ± 0.06 mL/g. The lowest value was observed for the control sample (CB with 2.07 ± 0.04). These results showed a minor loss of dough upon baking in the enriched formulations compared to the control bread, probably due to the high water retention capacity of the remaining mucilage in the expeller [34]. Previously, Silveira Coelho and de las Mercedes Salas-Mellado and coworkers [35] produced breads enriched with 11% of hydrated chia seeds, and they found that the enriched bread showed a significantly smaller volume than the control bread. This discrepancy was attributed to a decrease in the share of gluten in the bread’s dough due to a decreased ability to hold CO_2_ during fermentation and baking.

#### 3.3.3. Texture Profile Analysis

A detailed analysis of the textural properties (Table 1) showed no significant differences between the hardness parameters (H) of the CB, B1, and B3 bread formulations, while the values for B5 and B10 were considerably lower. This trend suggests that the addition of 5 and 10 mg of hydrolysates (with DH = 54.3 ± 1.6%) of chia expeller/g of flour may have improved the textural properties of the enriched bread compared to the control bread, resulting in a higher specific volume, lower firmness, and increase in the alveolate. In addition to changes in the Specific Volume (SV) and the hardness (H), bread fortification with 5 and 10 mg of hydrolysates also induced slight changes in the other textural properties of the bread, such as the resilience (R) and cohesiveness (Co) of the final baked product. In a previous report, Madruga et al. [5] reported a considerable reduction in the specific volume of the bread when 3 mg of chia seed hydrolysate (with DH = 30%/g of flour) was added to the bread formulation. The authors attributed these changes to a different formation of the “gluten network” due to the increase in the concentration of hydrolysate used in the formulation of the breads. In our study, the improvement in the textural parameters may be caused by the use of a chia expeller derivative with a superior degree of hydrolysis (DH = 54.3 ± 1.6%).

#### 3.3.4. Crumb Colour Analysis

Crumb colour is a key property of baked products since, together with volume and texture, it influences consumer acceptance. In our analysis, the crumb colour of the breads supplemented with hydrolysates from the chia expeller were expressed in CIE L*, a*, and b* values—corresponding to brightness, green (−)/red (+), and blue (−)/yellow (+), respectively—and compared to the standard bread (control). Table 2 summarises the colorimetric parameters obtained for the crumb of breads formulated with the chia expeller hydrolysates. In essence, no significant differences were observed in the brightness (L*) of breads B1, B3, and B5 compared to the control bread. Only breads with 10 mg of hydrolysate/g of flour showed a value of 69.39 ± 1.26, which is slightly lower compared to the control bread, which showed a value of 73.26 ± 0.74. This decrease in brightness can be attributed to the darker colour of the chia expeller hydrolysates compared to the wheat flour. However, the effect produced by baking represents only a 5% reduction compared to the control bread. A similar darkening effect was previously observed in another study after the addition of chia flour to the bread formulation [36,37,38].

The values of the a* and b* parameters showed a slight tendency towards reddish and yellow coloration in all the formulations, including the one with the highest concentration of chia expeller hydrolysates. Despite such small changes in coloration upon baking, no substantial differences were observed between the different bread formulations. Typically, the crumb colour of the bread crumbs tended to be yellow (h close to 90°). In this case, the value of the h parameter did not show significant differences between the CB (79.88 ± 0.65) and the bread formulation with the highest level of chia expeller hydrolysate supplementation, B10 (80.07 ± 0.17). Regarding the colour difference values (ΔE), the addition of different hydrolysate amounts slightly increased this parameter compared to the control bread. In summary, the results obtained for the chromatic parameters showed no considerable differences between the breads containing chia expeller hydrolysates and the control bread.

### 3.4. Antioxidant Activity of Baked Breads Fortified with Chia Expeller Hydrolysates

**Figure 3:**
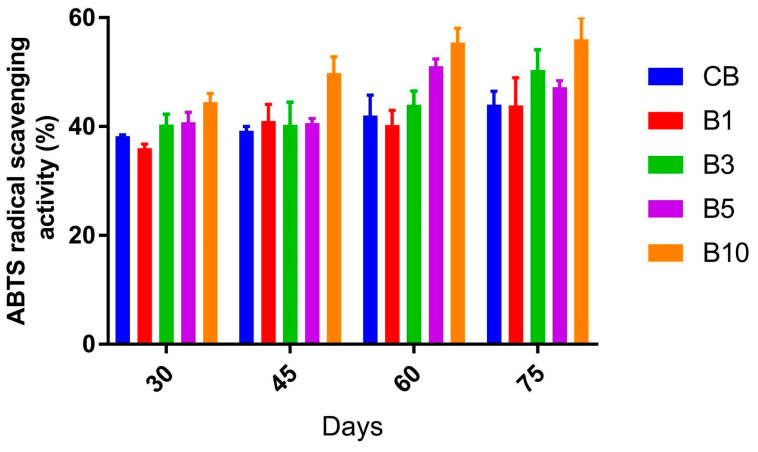
Antioxidant activity of breads supplemented with chia expeller hydrolysates. CB—Control bread; B1, B3, B5, and B10—Breads with 1, 3, 5, and 10 mg of chia expeller hydrolysate/g of flour.

The antioxidant activity of the different bread formulations was evaluated using the ABTS method after the breads were subjected to long-term storage (up to 75 days) at −20 °C. As shown in Figure 3 all fortified breads tested maintained or increased their antioxidant activity over time. Interestingly enough, the B10 bread formulation, which had 10 mg of chia expeller hydrolysate/g of flour, showed the highest antioxidant activity scores and a marked time-dependent increase in antioxidant capacity up to a value of 56 ± 3.9% after 75 days of storage at −20 °C. Under the same conditions, the control bread showed a much lower antioxidant activity (44 ± 2.5%). These results are consistent with those reported by Sayed-Ahmad et al. [37] and Romankiewicz et al. [39], who demonstrated that the addition of chia seed improved the antioxidant activity of wheat bread. Similar results were reported by Zdybel et al. [38], who evaluated the antioxidant potential against DPPH free radical scavenging of breads added with a waste product from the pressing of chia seed oil. In contrast, Segura-Campos et al. [40] demonstrated that the partial replacement of wheat flour by chia protein hydrolysates had no effect on the antioxidant activity of white bread, probably due to the effect of high temperatures during baking, causing the oxidation of tryptophan and histidine or the methionine desulphuration. However, in this study, the formulation contained only between 1 and 3 mg hydrolysates/g of flour.

In our study, the bread formulation supplemented with 10 mg of chia expeller hydrolysate/g of flour achieved increased antioxidant activity. Therefore, it is tempting to speculate that chia expeller hydrolysates could be a beneficial additive for improving the antioxidant properties of a novel generation of functional breads.

### 3.5. Sensory Test: Consumer Acceptability of Selected Bread Fortified with Chia Expeller Hydrolysate (B10)

**Figure 4:**
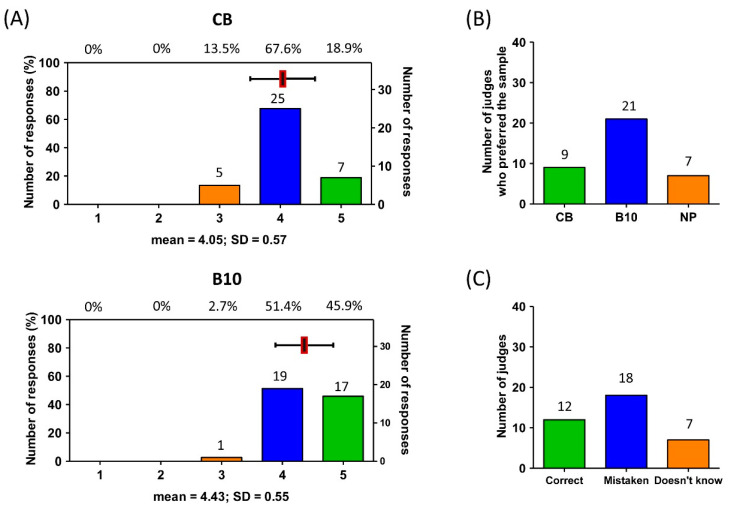
Sensory analysis of control breads (CB) and breads supplemented with 10 mg of chia expeller hydrolysate/g of flour (B10). (**A**) Five-point hedonic sensory evaluation. (**B**) Preference test. NP—No preference. (**C**) Triangular test. In (**A**), the mean (red square) ± Standard Deviation (SD) is displayed over the bars.

To gain insights into consumer preferences and acceptability criteria, consumer-oriented testing methods and untrained sensory panels have proven to be a valuable approach [41,42]. In order to assess the consumer acceptance and preference information for the formulations treated with chia protein hydrolysates in a wheat bread, the B10 formulation, which had highest amount of hydrolysate per gram of flour, was selected for a further preliminary sensory analysis [43]. This bread formulation showed a higher protein content and the best textural properties compared to the control bread. Moreover, this bread formulation exhibited a superior antioxidant capacity upon long-term storage. As summarised in Figure 4, the sensory analysis of the breads supplemented with 10 mg of hydrolysate/g of flour, 17 untrained panellists awarded the B10 formulation with the best possible rating (“I liked it very much”), while 19 panellists gave it the second-best possible rating, stating “I liked it”. Compared to the control wheat bread, where the rating of “I liked it” predominated, a positive trend was found regarding the bread fortified with 10 mg of hydrolysate, which received the best score (Figure 4A). In a similar manner, when carrying out the preference test (Figure 4B), 21 panellists chose bread B10 over the control bread (which was preferred by 9 panellists), while 7 panellists did not present a preference for any particular bread. Out of the 37 panellists, 24 positive responses had to have been obtained for one of the samples to ensure that there was a significant difference (*p* < 0.05). Therefore, from the 21 positive responses received for the bread fortified with chia hydrolysates, it can be inferred that the addition of 10 mg of hydrolysates/g of flour did not show significant differences in the preferences of the panellists with respect to the control bread.

In a recent study, Madruga and coworkers [5] prepared wheat and rice breads supplemented with chia hydrolysates. In this study, the authors concluded that the addition of chia hydrolysate flour did not affect the consumer preference. The similarities in the observations between our work and previously reported work could be due to the reduced impact of sensory attributes introduced by the addition of hydrolysates at the tested concentration. On the contrary, a previous study by da Silva et al. [44] found that the addition of *ora-pro-nobis* (*Pereskia aculeata*) to salt bread had a substantial impact on consumer preference. In a similar manner, Robles-Ramírez et al. [45] recently reported that the substitution of wheat flour with 60% barley flour caused a reduction in consumer preference. Interestingly, the sensory qualities of this bread were improved by adding sesame seeds to the bread formulation.

Finally, a triangular test was also carried out to investigate whether the panellists were able to identify fine differences between the three bread samples. As shown in Figure 4C, only 12 panellists were able to identify the different bread samples, while 18 of them pointed out an erroneous sample as different, and 7 of them found no differences. These results are in agreement with the preference test since there were no significant differences between the preference of the panellists towards a particular sample.

Taken together, the results from the sensory evaluation suggest that both the control and fortified breads present similar consumer preferences and overall sensory characteristics since consumers could not identify which one corresponded to the formulation fortified with the chia expeller hydrolysates.

## 4. Conclusions

This study represents the first attempt to develop a wheat bread product enriched with functional peptides derived from the chia expeller. For this purpose, we first aimed to prepare a protein hydrolysate from defatted chia expeller via enzymatic hydrolysis with Alcalase, reaching a degree of hydrolysis of 54.3 ± 1.6% after 6 h of hydrolysis at 25 °C. This chia expeller hydrolysate (PCH25,6) showed a notable antioxidant activity of 55.8 ± 0.4%, as determined via the ABTS method. Moreover, the proximate composition values of the PCH25,6 hydrolysate suggested that this additive contains a notable amount of protein and fibre, which is comparable to the values found in raw chia seeds. In this context, the techno-functional characterization of the hydrolysate showed adequate values of emulsifying activity, as well as water and oil retention capacities, rendering it compatible as a supplemental component in bread formulations.

Notably, the fortification of wheat breads with different amounts of chia expeller hydrolysates (i.e., 1, 3, 5, and 10 mg of chia expeller hydrolysate/g of flour) did not affect the bread’s textural properties and provided a higher antioxidant activity even after 75 days of storage at −20 °C. The most substantial enhancement in antioxidant activity was observed when 10 mg of chia expeller hydrolysate was added to the bread formulation.

More importantly, the bread prepared with the highest content of hydrolysates showed acceptable sensorial attributes that were comparable to the control bread. In a preliminary consumer study, the participants enjoyed trying the bread product supplemented with the chia expeller hydrolysates, but no significant differences in consumer preference were identified between the control and fortified bread samples. This finding is in agreement with the results from a previous study by Madruga and coworkers [5], in which the authors demonstrated that the sensory characteristics of wheat and rice bread supplemented with 3 mg of chia hydrolysate/g flour were comparable to those of the control bread.

Taken together, the results presented herein demonstrate that the incorporation of chia expeller hydrolysates into a novel wheat bread formulation could be a valuable way of increasing the protein content of bread. Furthermore, it positions the fortified bread as a functional wheat bread product that offers increased protein content and enhanced antioxidant activity. Ultimately, this approach facilitates the utilisation of a by-product derived from the chia oil industry, offering both significant economic and environmental advantages.

## Figures and Tables

**Table 1:** Technological characteristics of breads supplemented with different concentrations of chia expeller hydrolysates compared to wheat flour.

Bread	SV (mL/g)	H (g)	A (g. sec)	R (%)	Co	S (%)	G	Ch
**CB**	2.07 ± 0.04	2986.4 ± 936	−0.90 ± 0.60	15.31 ± 2.34	0.44 ± 0.04	88.03 ± 2.48	1304.2 ± 357	1145.1 ± 306
**B1**	2.11 ± 0.03	2696.2 ± 546	−0.83 ± 0.61	15.11 ± 1.24	0.44 ± 0.03	86.76 ± 2.95	1170.3 ± 194	1016.2 ± 175
**B3**	2.25 ± 0.06 ^b^	2478.5 ± 687	−0.71 ± 0.61	15.71 ± 1.67	0.45 ± 0.03	87.87 ± 2.09	1116.6 ± 297	981.2 ± 261
**B5**	2.29 ± 0.08 ^b^	2197.4 ± 449	−0.32 ± 0.57	18.89 ± 2.45	0.50 ± 0.04	88.51 ± 1.62	1086.2 ± 180	960.4 ± 154
**B10**	2.27 ± 0.05 ^b^	2160.9 ± 342	−0.59 ± 0.62	19.16 ± 1.87	0.51 ± 0.02	91.05 ± 2.63	1102.1 ± 153	1003.0 ± 140

Abbreviations are as follows: CB—Control bread; B1, B3, B5, and B10—Breads supplemented with 1, 3, 5, and 10 mg of chia expeller hydrolysate/g of flour; SV—Specific volume; H—Hardness; A— Adhesiveness; R—Resilience; Co—Cohesion; S—Springiness; G—Gumminess (G = H × Co); Ch— Chewiness (Ch = G × S = H × Co × S). Values show the average of three values ± the standard deviation (SD). ^b^ indicates a significant difference between the means (*p* < 0.05; Tukey test).

**Table 2:** Colour parameters of the crumbs of breads supplemented with chia expeller hydrolysates.

Bread Formulation	L*^2^	a*^2^	b*^2^	h (°)	ΔE
**CB**	73.26 ± 0.74	4.88 ± 0.39	27.33 ± 0.39	79.88 ± 0.65	0.92
**B1**	73.07 ± 0.50	4.13 ± 0.13	26.40 ± 0.59	81.11 ± 0.08	0.78
**B3**	73.54 ± 1.05	4.27 ± 0.10	26.41 ± 0.18	80.82 ± 0.15	1.07
**B5**	71.12 ± 1.19	4.68 ± 0.28	26.94 ± 0.33	80.15 ± 0.46	1.27
**B10**	69.39 ± 1.26 ^b^	4.58 ± 0.13	26.15 ± 0.29	80.07 ± 0.17	1.30

Abbreviations are as follows: CB—Control bread; B1, B3, B5, and B10—Breads supplemented with 1, 3, 5, and 10 mg of chia expeller hydrolysate/g of flour. L*—Lightness; a*—Tendency for green–red coloration; b*—Tendency for blue–yellow coloration; h—Hue angle; ΔE—colour difference values. Values show the average of three values ± the standard deviation (SD). ^b^ indicates a significant difference between the means (*p* < 0.05; Tukey test).

## Data Availability

The data used to support the findings of this study can be made available by the corresponding author upon request.

## Supplementary Materials

The following supporting information can be downloaded at: https://www.mdpi.com/article/10.3390/foods12183382/s1, Figure S1: Process flow diagram for obtaining and characterizing chia expeller protein hydrolysates; Table S1: Summary of the different bread formulations prepared in this study; Table S2: Proximate composition of chia expeller hydrolysates produced with Alcalase at 25 °C for 6 h (PCH25,6) compared to chia seeds; Table S3: Techno-functional characterization of chia expeller hydrolysates produced with Alcalase at 25 °C for 6 h (PCH25,6).
