# Supplementary material for: Development of Fortified Breads Enriched with Plant-Based Bioactive Peptides Derived from the Chia (Salvia hispanica L.) Expeller
Source: Foods. 2023 Sep 9;12(18):3382. doi: 10.3390/foods12183382 (PMC10528513; doi:10.3390/foods12183382)
Supplement: Supplementary file 1 [file foods-12-03382-s001.zip › foods-2588945-supplementary.pdf]

**SUPPLEMENTARY INFORMATION**

# Development of Fortified Breads Enriched with Plant-Based Bioactive Peptides derived from the Chia (Salvia hispanica L.) Expeller

Brenda Ozón et al.

Supplementary Figure S1 and Supplementary Tables S1, S2 and S3

**Figure S1**

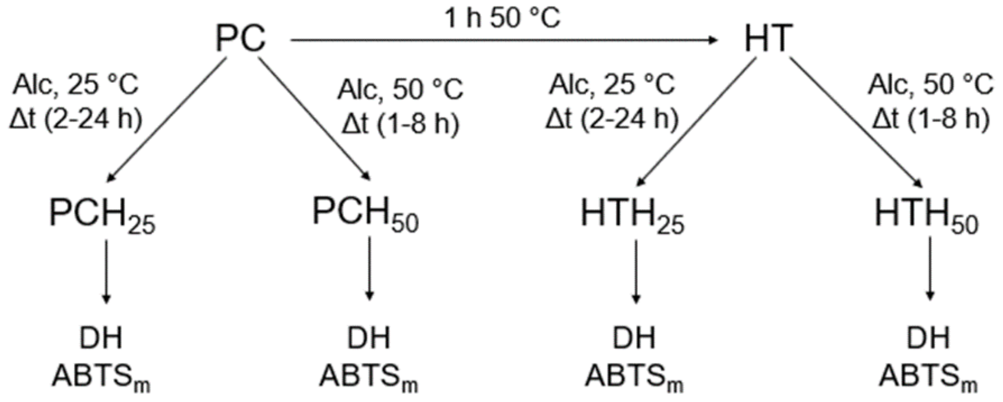


**Figure S1. Process flow diagram for obtaining and characterizing chia expeller protein hydrolysates.** PC: Chia expeller protein concentrate; HT: Heat treated; Alc: Alcalase; DH: Degree of hydrolysis; ABTSm: Antioxidant activity by ABTS method; PCH25 samples: PC hydrolysate at 25 °C for 2, 4, 6, 8, 10 and 24 h; PCH50 samples: PC hydrolysate at 50 °C for 1, 2, 3, 4, 5, 6, 7 and 8 h; HTH25 samples: HT hydrolysate at 25 °C for 2, 4, 6, 8, 10 and 24 h; HTH50 samples: HT hydrolysate at 50 °C for 1, 2, 3, 4, 5, 6, 7 and 8 h.

## Table S1

**Table S1.** Summary of the different bread formulations prepared in this study.

| Ingredients | CB | B1 | B3 | B5 | B10 |
|---|---|---|---|---|---|
| **Water (mL)** | 100 | 100 | 100 | 100 | 100 |
| **Milk (mL)** | 200 | 200 | 200 | 200 | 200 |
| **Butter (g)** | 50 | 50 | 50 | 50 | 50 |
| **Sugar (g)** | 30 | 30 | 30 | 30 | 30 |
| **Dry yeast (g)** | 13 | 13 | 13 | 13 | 13 |
| **Wheat flour (g)** | 500 | 500 | 500 | 500 | 500 |
| **Salt (g)** | 2 | 2 | 2 | 2 | 2 |
| **PCH25,6 (g)** | 0 | 0.5 | 1.5 | 2.5 | 5 |

Abbreviations are: CB, Control bread; B1, B3, B5 and B10, Breads added with 1, 3, 5 and 10 mg of hydrolysate/g of flour respectively; PCH25,6, Hydrolysates produced with Alcalase for 6 h at 25°C from chia expeller.

## Table S2

**Table S2.** Proximate composition of chia expeller hydrolysates produced with Alcalase at 25 °C for 6h (PCH25,6) compared to chia seeds.

| | | Reference values from [26] |
|---|---|---|
| **Components** | **(%)** | **(%)** |
| **Moisture** | 9.6 | ND |
| **Ashes** | 33.2 | 4.8 |
| **Lipids** | 1.6 | 30.7 |
| **Proteins** | 19.5 | 16.5 |
| **Carbohydrates (dietary fibre)** | 30.2 | 34.4 |
| **Assimilable Carbohydrates** | 5.9 | 7.7 |

Abbreviations are: ND, Not Determined

## Table S3

**Table S3.** Techno-functional characterization of chia expeller hydrolysates produced with Alcalase at 25 °C for 6h (PCH25,6).

| Properties | $PCH_{25,6}$ | Villanueva-Lazo et al [29] |
|---|---|---|
| **Water retention capacity** (g/g of hydrolysate) | 1.92 ± 0.08 | ND |
| **Oil retention capacity** (g/g of hydrolysate) | 2.36 ± 0.35 | 3.5± 0.05 |
| **Water adsorption capacity** (g/g of hydrolysate) | 0.59 ± 0.03 | ND |
| **Emulsifying activity** (mL/100 mL) | 41.26 ± 2.80 | 50.3± 0.05 |
| **Emulsion stability** (mL/100 mL) | 69.70 ± 0.35 | 89.8 ± 0.05 |

Values are means ± SD of triplicate determinations. Abbreviations are: ND, Not Determined
